# Prediction and analysis of multi epitope based vaccine against Newcastle disease virus based on haemagglutinin neuraminidase protein

**DOI:** 10.1016/j.sjbs.2022.01.036

**Published:** 2022-01-20

**Authors:** Adnan Raza, Muhammad Asif Rasheed, Sohail Raza, Muhammad Tariq Navid, Amna Afzal, Farrukh Jamil

**Affiliations:** a Department of Biosciences, COMSATS University Islamabad, Sahiwal Campus, Sahiwal, Pakistan; b Institute of Microbiology, University of Veterinary and Animal Sciences, Lahore, Pakistan; c Department of Biological Sciences, National University of Medical sciences, Rawalpindi 46000, Pakistan

**Keywords:** Epitopes, Newcastle Disease Virus, Paramyxovirus Virus, Vaccine

## Abstract

Newcastle disease virus (NDV), an avian orthoavulavirus, is a causative agent of Newcastle disease named (NDV), and can cause even the epidemics when disease is not treated. Previously several vaccines based on attenuated and inactivated viruses have been reported which are rendered useless with the passage of time due to versatile changes in viral genome. Therefore, we aimed to develop an effective multi-epitope vaccine against the haemagglutinin neuraminidase (HN) protein of 26 NDV strains from Pakistan through a modern immunoinformatic approaches. As a result, a vaccine chimaera was constructed by combining T-cell and B-cell epitopes with the appropriate linkers and adjuvant. The designed vaccine was highly immunogenic, non-allergen and antigenic; therefore, the potential 3D-structureof multi epitope vaccine was constructed, refined and validated. A molecular docking study of a multiepitope vaccine candidate with the chicken Toll-like receptor-4 indicated successful binding. An *In silico* immunological simulation was used to evaluate the candidate vaccine's ability to elicit an effective immune response. According to the computational studies, the proposed multiepitope vaccine is physically stable and may induce immune responses whichsuggested it a strong candidate against 26 Newcastle disease virus strains from Pakistan.

## 1 Introduction

Newcastle disease is a highly contagious disease that affects a wide range of domestic and wild bird species. It was initially reported in 1926 from Java, Indonesia, and Newcastle-upon-Tyne, England, but there are earlier records of comparable epidemics in central Europe (Osman et al. 2016). When the first release of avian pathology came out in 1972, the poultry industry faced a devastating outbreak throughout the world. Within two decades of its discovery in United Kingdom and Indonesia in 1926, the disease had reached pandemic proportions. Currently, the disease is present in many countries throughout the world (Dimitrov et al. 2016).

Newcastle disease is caused by the strains of avian paramyxovirus type 1 (APMV-1) (Dimitrov et al. 2016). Newcastle disease virus (NDV) is one of the specie of the Avulavirus genus, order Mononegavirales, family Paramyxoviridae. NDV was newly identified as member of the Genus Avian Orthoavulavirus, Family Paramyxoviridae, and is now designated as Avian Orthoavulavirus-1 (AOAV-1) (El-hamid et al. 2020).

NDV is mostly spread through direct contact by body exposure of infected birds. The virus has been identified in all regions of the infected body and may survive for months on chicken skin and bone marrow. Depression, loss of appetite, unusual thirst, acute thirst, paralysis, and fever are all common symptoms of ND. As the virus is stable outside the host and in the environment, fomite transmission is possible. The contagious virus is found in infected chicken corps for 7 days in the summer, 14 days in the spring, and 30 days in the winter (Kinde et al. 2004).

Moreover, the death rate can exceed 100 percent (Alexander et al. 2012). Mesogenic virus can cause clinical disease that usually involves respiratory and neurological symptoms but infection is limited and death is rare in older birds. Currently, 15 avian paramyxovirus serotypes (APMV-1 to APMV-15) have been identified in diverse species of wild and domestic birds, where they have been linked to respiratory illnesses and a considerable decrease in egg production (Lugarini et al. 2018). APMV-1 strains are phylogenetically and genetically separated into two main classes, class I and class II. Among these classes, class I members are assigned to only one genotype while class II subdivisions are further differentiated into genotypes I-XVIII, all of which are expected to be virulent in hens, with the exception of those assigned to genotypes I, II, and X (Dimitrov et al. 2016).

Morphologically, NDV virions are 100 nm or more wide, pleomorphic, but mostly round in shape (Schirrmacher 2017). These sequence encoding for six proteins, i.e., phosphoprotein (P), haemagglutinin neuraminidase (HN), nucleoprotein (NP), large protein (L), fusion protein (F)andmatrix protein (M). NDV appears to be a pleomorphic-coated molecule containing F and HN spike glycoproteins that participate in the onset of the infectious cycle. HN consists of 577 residues (Phale 2018), and it involves in cell adhesion (Dimitrov et al. 2019). Moreover, it releases two non-structural proteins, V and W (Murulitharan et al. 2013). While, M protein is located immediately beneath the virus envelopewhich is known to help in maintaining viral form and aid in the packaging and release of virions (Bello et al. 2018).

Vaccination is widely regarded as the most efficient method to prevent the infectious diseases (Hellstrom and Hellstrom 2003). There are two ways to ND vaccination strategies: traditional methods introduced in the 1940 s and newly developed methods based on duplicate DNA technology.

Many type of NDV strains have been used to vaccinate against ND in the poultry industry (Hanson 1955). Number of lentogenic NDV strains such as B1, F, LaSota, V4, and I2 are extensively used as live vaccines for disease control (Commission 2018). Both live and inactivated vaccines, have been regularly utilized since 1950 s. Some countries use recombinant and antigenically matched vaccines, whereas many other vaccination techniques have just been tested experimentally. For example a recombinant ND vaccine “Innovax-ND” was approved by the US FDA for commercial use (Schirrmacher 2017). Despite decades of research and development aimed at developing an ideal vaccine, improvements are still required (Kiril, Claudio, and Patti 2016). Advance information of antigen acknowledgment at the cellular level has contributed to the advancement of well-designed peptide antibodies. The common principle of peptide vaccinations is based on the chemical approach of mixing immunodominant B-cell and T-cell epitopes that can produce specific immunity. The B-cell epitopes can be coupled with the T-cell epitopes to make it less viable. The researchers developed the first epitope based vaccine in 1985 (Jacob et al. 1985). T-cell epitopes are peptide fragments, whereas B-cell epitopes are proteins, lipids, nucleic acids, or carbohydrates (Dermime et al., 2004, Lehner et al., 1990, Mahleret al., 2003, Meloenet al., 2001). Furthermore, certain multi-epitope vaccinations have already started phase I clinical trials (Lennerz et al. 2014). Computational modeling of multi-epitope vaccines against parasites, virus, bacteria, and even cancer has become prevalent recently (Shey et al. 2019). Multiepitope vaccines have several advantages over conventional (i.e., live and attenuated) vaccines. Moreover, it can be altered in a variety of ways, such as merging T- and B-cell epitopes, removing unwanted components, adding adjuvant, and so on. As a result, a well-designed multi-epitope vaccination could serve as an effective preventative drug (Saadi, Karkhah, and Nouri 2017) against Newcastle disease virus.

In this study the goal of work is to use a set of immunoinformatics methods to develop a multi-epitope vaccine based on HN protein against Newcastle disease virus. HN protein is the most antigenic protein in NDV proteome and utilized in current study to predict T- and B-cell epitopes, which was followed by multi-epitope vaccine development. The candidate vaccine may be helpful to trigger the immunity against NDV and protect poultry from contagious disease.

## 2 Material and methods

### 2.1 Retrieval of viral proteome and antigenicity prediction

The complete amino acid sequences of the total six Newcastle disease virus proteins of the strain of Pakistan including hemagglutinin-neuraminidase (Accession no. QXI73423.1), fusion protein (Accession no. QXI73422.1), matrix protein (Accession no. QXI73421.1), phosphoprotein (Accession no. QXI73420.1), nucleocapsid protein (Accession no. QXI73419.1) and large protein (Accession no. QXI73424.1) were obtained in the FASTA format from NCBI (https://www.ncbi.nlm.nih.gov/). To detect the potential antigenicity of the NDV proteins, a web predictive server, VaxiJen v2.0 (http://www.ddg-pharmfac.net/vaxijen/VaxiJen/VaxiJen.html) was utilized to predict antigenicity of each protein. Moreover, we collected the protein sequences of all NDV strains of Pakistan from NCBI Virus database.

### 2.2 Consensus sequence

We used Geneious Prime software to do the multiple sequence alignment and from it we determined consensus sequence. Multiple sequence alignment was done with global alignment and Blosum62 scoring matrix algorithm.

### 2.3 Prediction of CTL epitopes

The NetCTL v1.2 server at (https://www.cbs.dtu.dk/services/NetCTL/) was used to predict the presence of any human CTL (cytotoxic T-lymphocytes) epitopes in the chosen protein with default 0.75 threshold score (Larsen et al. 2007). Chicken MHC alleles in immunoinformatics online tools were unavailable so, previous investigations employed human MHC alleles to predict epitopes. Chicken BF alleles have been demonstrated to stimulate an immunological response similar to human class I homologous alleles, notably in antigen presentation (Mugunthan and Harish 2021). So, this server was used to predicted 9-mer length CTL epitopes at default values. This is accomplished by combining three features, namely, TAP transport efficiency, C-terminal cleavage and MHC-I binding peptides (Larsen et al. 2007). Furthermore, to select the final CTL epitopes, they were screened by using VaxiJen v2.0, AllerTOP v.2.0, and ToxinPred servers.

### 2.4 Prediction of HTL epitopes

The NetMHCIIpan 4.0 server at (http://www.cbs.dtu.dk/services/NetMHCIIpan/) was used to predict 15-mer long Helper T-lymphocytes (HTL) epitopes within the chosen protein with default parameters. The NetMHCIIpan 4.0 serverwas affiliated with Class II human leukocyte antigen (HLA) pairings (K. K. Jensen et al. 2018). Furthermore, in addition to select the final HTL epitopes, they were screened byusing VaxiJen v2.0, AllerTOP v.2.0, and ToxinPred servers as well.

### 2.5 Prediction of B-cell epitopes

The immune systemmost significant component is B lymphocytes. It is in charge of secreting antibodies, which give long-term immunity (J. Zhang et al. 2014). We used BCPred (http://crdd.osdd.net/raghava/bcpred/) server for the identification of continuous 20-mer longB-cell lymphocyte (BCL) for the chosen proteinwith threshold value < 8.0 (Kumar et al. 2021) and in addition to select the final linear BCL epitopes, we used VaxiJen v2.0, AllerTOP v.2.0, and ToxinPred servers as well.

### 2.6 Construction of vaccine

The selected CTL, HTL and BCL epitopes were connected together by using appropriate linkers. The linkers are selected due to their two features. Firstly, they prevent junctional epitopes formation, and secondly, they improve epitope presentation (Khan et al. 2019). Each CTL epitope was joined with AAY linkers, whereas HTL and BCL were joined with GPGPG and KK linkers (Nain et al. 2020), respectively. Additionally, 65 amino acid long avian beta defensin (AvBD) (GenBank accession number: NP_990324) was linked to the 5′ terminus of vaccine as an adjuvant by utilizing the EAAAK linker to increase the immunogenicity of vaccine as mentioned in (Khan et al. 2019).

### 2.7 Allergenicity and antigenicity

In order to predict the allergenicity of vaccine construct we used an online server called AllerTOP (https://www.ddg-pharmfac.net/AllerTOP/) to make sure that the construct vaccine didn't cause any allergic reactions. Furthermore, antigenicity testing is a crucial part of the vaccine development process so, VexiJen 2.0 (http://www.ddg-pharmfac.net/vexijen/) an online web server was used to predict the antigenicity of our vaccine construct with threshold value 0.4 (Doytchinova and Flower 2007).

### 2.8 Physicochemical properties evaluation

Expasy-ProtParam server (http://www.expasy.org/protparam/) was used to evaluate numerous physicochemical aspects of the vaccine sequence. Amino acid composition, protrusion index (PI), half-life, aliphatic score, instability index, molecular weight and GRAVY (Grand average of hydropathicity index) of the vaccine, all of these aspects was calculated by using ProtParam this server (John 2005).

### 2.9 Structure prediction, refinement and validation

The 3D model of the primary vaccine construct sequence was predicted using trRosetta (https://yanglab.nankai.edu.cn/trRosetta/). It builds the protein structure using direct energy minimizations. (Yang et al. 2020). 3Drefine (http://sysbio.rnet.missouri.edu/3Drefine/) a protein structure refinement server was used to refine the overall structure quality of the predicted 3D model. After refinement, the RAMPAGE server was used to evaluate the overall quality of the refined vaccine model (Lovell et al. 2003). Ramchandran plot validate the construct’s structure by energetically favored and unfavored dihedral angles i.e., psi (Ψ) and phi (Φ) of amino acid residues (Laskowski et al. 1993).

### 2.10 Docking

It is critical for the vaccine to engage with target immune cell receptors in order to generate a persistent immunological response. Molecular docking experiments are used to investigate such interactions (Nawab et al. 2019). As a result, a molecular docking analysis was carried out to evaluate the MEV's interaction with the chicken immune receptor (ChTLR4). Scientists have discovered that chTLR4 plays an important role in the production of both innate and adaptive immune responses in chicken (G. Zhang and Sunkara 2014). So, docking of our designed MEV with TLR4 (PDB ID: 3mu3) was performed by using PatchDock (https://bioinfo3d.cs.tau.ac.il/PatchDock/). The PatchDock method is used to predict protein–protein and protein–small molecule complex interactions (Duhovny, Nussinov, and Wolfson 2002). Moreover, FireDock web server (https://bioinfo3d.cs.tau.ac.il/FireDock/) was used for further electrostatic interaction scoring and refinement to get the best complex (Mashiach et al. 2008).

### 2.11 Vaccine construct immune simulation

An *In silico* immune simulation was performed using the C-IMMSIM server available at (http://150.146.2.1/C-IMMSIM/index.php) to validate the immunological response of the designed vaccine (Rapin et al. 2010). This server simulates three major components of a working mammalian system (lymph node, bone marrow and thymus). In practical practice, a four-week (28 days) interval between 2 vaccine doses is recommended (Rapin et al. 2010). The simulation step is the key parameter in C-IMMSIM server. One simulation step equals eight hours. So, in the first round, we set the simulation step value to 100 in order to monitor the effect of the vaccine for a total of 35 days, and in the second round, we fixed it to 84. So, by setting it to 84 the second dose will be administered after 28 days following the first dose.

## 3 Results

### 3.1 Sequence retrieval and selection of most antigenic protein

The VexiJen v2.0 server analysis revealed that both haemagglutinin neuraminidase (HN) and fusion (F) protein were the most antigenic proteins with the same antigenicity score as compared to other 4 proteins of NDV (Table 1). So, we chose HN protein for our further analysis. HN protein is 577 amino acids long with antigenic score 0.559 which is 1.4 times higher than server threshold value 0.4 that suggested it a strong candidate for multi epitope vaccine construct. Moreover, we selected a total of 26 strains of NDV in the NCBI Virus database under the Pakistan geographic region and downloaded haemagglutinin neuraminidase (HN) protein sequences from all selected strains. Consensus sequence analysis revealed that only 571 out of 577 amino acids of HN protein were conserved among all the 26 strains that suggested very little variance. As a result, the vaccine developed against one strain can be utilized among all other 25 strains of NDV.

**Table 1:** List of Newcastle disease virus proteins.

No.	Protein name	Antigenecity score	No. of amino acids
1.	Hemagglutinin-neuraminidase (HN)	0.559*	577
2.	Fusion glycoprotein (F)	0.559	553
3.	Matrix protein (M)	0.514	364
4.	Phosphoprotein (P)	0.468	395
5.	Large protein (L)	0.439	2204
6.	Nucleoprotein (NP)	0.392	489

*Selected protein for multi epitope-based vaccine construction.

### 3.2 Prediction of T cell epitopes

NetCTL1.2 server predicted 21 CTL epitopes within the HN protein consensus sequence with prediction score < 0.75. While NetMHC II pan 4.0 server predicted 3 HTL epitopes which have strong threshold value (1% default score) for binding peptides. Only 7 CTL epitopes out of 21 and 1 HTL epitope out of 3 were showed antigenic, non-allergen and non-toxic nature. So, as a result, 7 CTL epitopes and 1 HTL epitope were selected for the chimaera construction which were summarized in (Table 2) and (Table 3) respectively.

**Table 2:** Selected cytotoxic T-lymphocytes **(**CTL) epitopes predicted by using NetCTL 1.2 server.

**No.**	**Epitopes**	**Position**	**Prediction score**	**Antigenicity**	**Allergenicity**	**Toxicity**
1.	AISAAALAY	38	2.1491	0.8318	Non-allergen	Non-toxin
2.	VTSFYPSAY	148	3.1560	0.4130	Non-allergen	Non-toxin
3.	TVGTSHFLY	406	2.1275	0.5265	Non-allergen	Non-toxin
4.	SYFSPALLY	419	0.7882	0.6322	Non-allergen	Non-toxin
5.	HSPYTFNAF	439	0.7520	0.4646	Non-allergen	Non-toxin
6.	YTDPYPLIF	470	3.5092	0.9134	Non-allergen	Non-toxin
7.	SSSSTKAAY	518	2.5277	0.7846	Non-allergen	Non-toxin

**Table 3:** Selected helper T-lymphocytes **(**HTL) epitope predicted by NetMHC II pan 4.0 server.

**No.**	**Epitope**	**Position**	**Prediction Score**	**Antigenicity**	**Allergenicity**	**Toxicity**
1.	KVFFSTLRSINLDDT	218	1.00	0.7151	Non-allergen	Non-toxic

### 3.3 Prediction of B cell epitopes

BCPredserver v 1.0 predicted 12 linear B-cell epitopes with threshold value < 8.0 which were further analyzed for antigenicity and allergenicity and toxic nature. Only 4 epitopes out of 12 showed antigenic, non-allergen and non-toxic nature which were summarized in (Table 4).

**Table 4:** Selected B cell epitopes prediction by BCPred server v1.0.

**No.**	**Epitope**	**Position**	**Prediction score**	**Antigenicity**	**Allergenicity**	**Toxicity**
1.	SKVTETEEEDYKSVTPTSMV	252	0.98	0.9297	Non-Allergen	Non-toxin
2.	PVYGGLKPNSPSDTAQEGKY	315	0.945	0.5997	Non-Allergen	Non-toxin
3.	ASARCPNSCITGVYTDPYPL	457	0.914	0.7575	Non-Allergen	Non-toxin
4.	DDGQARLNPVSAVFDDISRS	493	0.851	0.5468	Non-Allergen	Non-toxin

### 3.4 Multi epitope vaccine construct

**Fig. 1:**
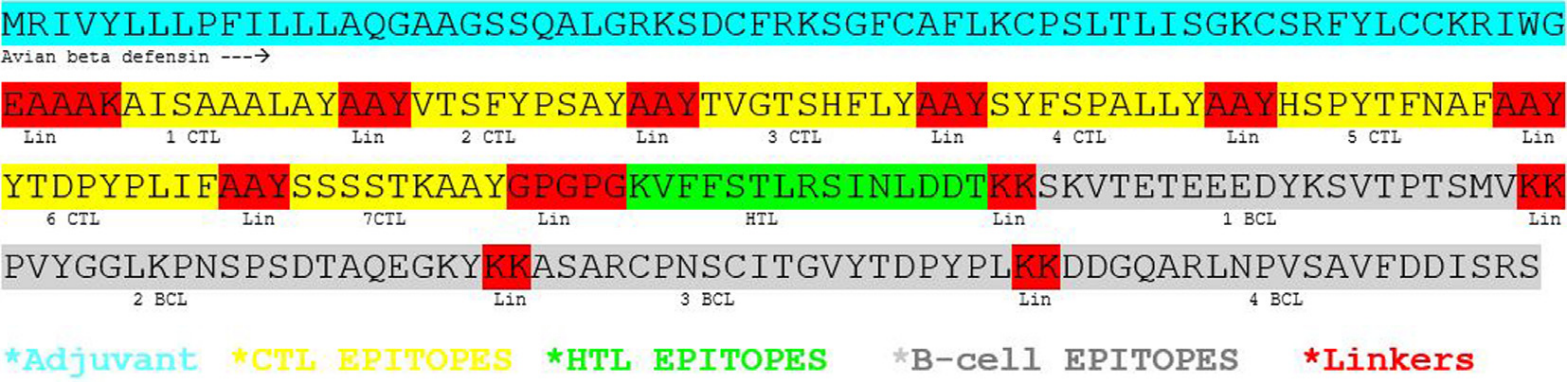
Multi-epitope vaccine construct of HN protein.

To construct multi-epitope-based vaccine, the selected7 CTL,1 HTL and 4B-cell epitopes were joined by using AYY, GPGPG and KK linkers (Fig. 1). Avian beta defensin as an adjuvant protein was connected to the N-terminal of first CTL epitope by using EAAAK linker.Remaining 6 CTL epitopes were joined together through AAY linkers while the last CTL was joined with HTL epitope though GPGPG linker. Furthermore, HTL epitope was linked with first BCL epitope through KK linker and remaining 3 BCL epitopes were further joined together by using KK linker.

### 3.5 Physiochemical properties

Various physiochemical properties of our designed vaccine were shown in Table 5. The multi-epitope vaccine contains 259 amino acids and has a molecular weight of 28.18 KDa. The physiochemical PI value was found to be 9.21. The vaccine has an aliphatic index (AI) of 71.0. According to ExPASy-ProtParam server, our designed vaccine expected half-life was 30 h in mammalian reticulocytes (*In vitro*) >20 h in yeast (*In vivo*) and over 10 h in *E. coli (In vivo)*. The GRAVY score was-0.087 while vaccine candidate instability index (II) was found to be 39.14. Moreover, antigenicity score of our vaccine was 0.5214 as predicted by VaxiJen v2.0 server and by AllerTOP our vaccine was confirmed to be non-allergen. This implies that the designed vaccine is immunogenic and capable of eliciting an adequate immune response.

**Table 5:** Physiochemical properties of HN protein based multi-epitope vaccine.

**Parameter**	**Results**	**Remarks**
Number of amino acids	259	Suitable
Molecular weight	28.18KDa	Average
Theoretical pI	9.21	significantly basic
Ext. coefficient	40,270	
Estimated half-life	30 h (mammalian reticulocytes, in vitro) >20 h (yeast, in vivo) >10 h (Escherichia coli, in vivo)	Satisfactory
Instability index	39.14	Stable
Aliphatic index	71.00	Thermostable
Grand average of hydropathicity (GRAVY)	−0.087	Hydrophilic

### 3.6 Structure prediction, refinement and evaluation

**Fig. 2:**
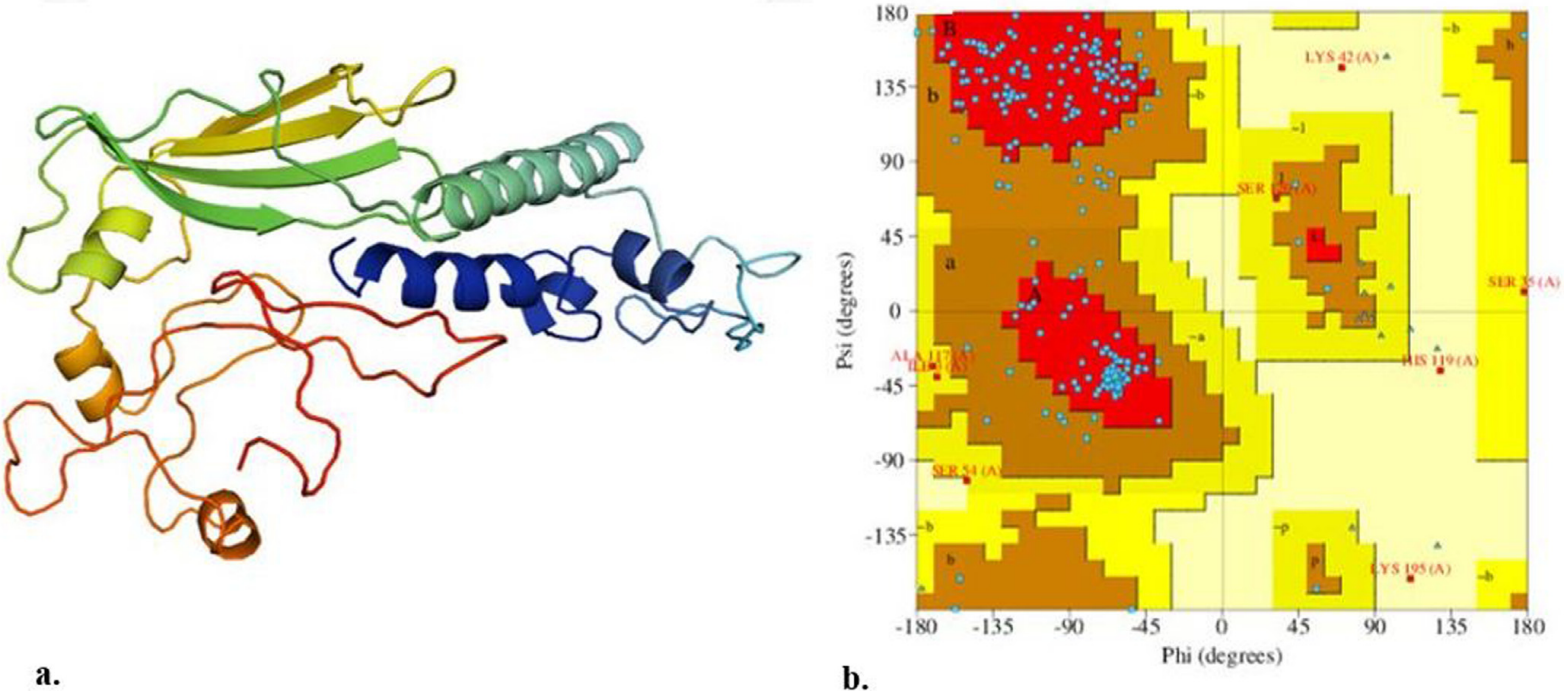
(a)Refined3Dstructure of vaccine construct; (b) Ramachandran plot analysis of vaccine construct using PROCHECK.

trRosetta predicted a 3D protein structure which was consisted on 7 helices and 4 beta sheets. The structure was further subjected to3D refine server. The output gives 5 refined 3D models of the vaccine construct. The model number 1 was found to be the best refined model having good RMSD (0.140), GDT-HA (1.00), GDT-TS (1.00) and MolProbity (2.490) as compared to other models (Fig. 2**a**). Ramachandran plot (Fig. 2**b**) study of the refined 3D-model revealed that 78.2% of residues were in the most preferred region, 18.2% residues in additional allowed region, 1.8% residues in generously allowed region and 1.8% residues in disallowed region, respectively indicating that the vaccine's overall quality was good (Laskowski et al. 1993). So, this model was used for further analysis.

### 3.7 Docking of vaccine construct with TLR4

**Fig. 3:**
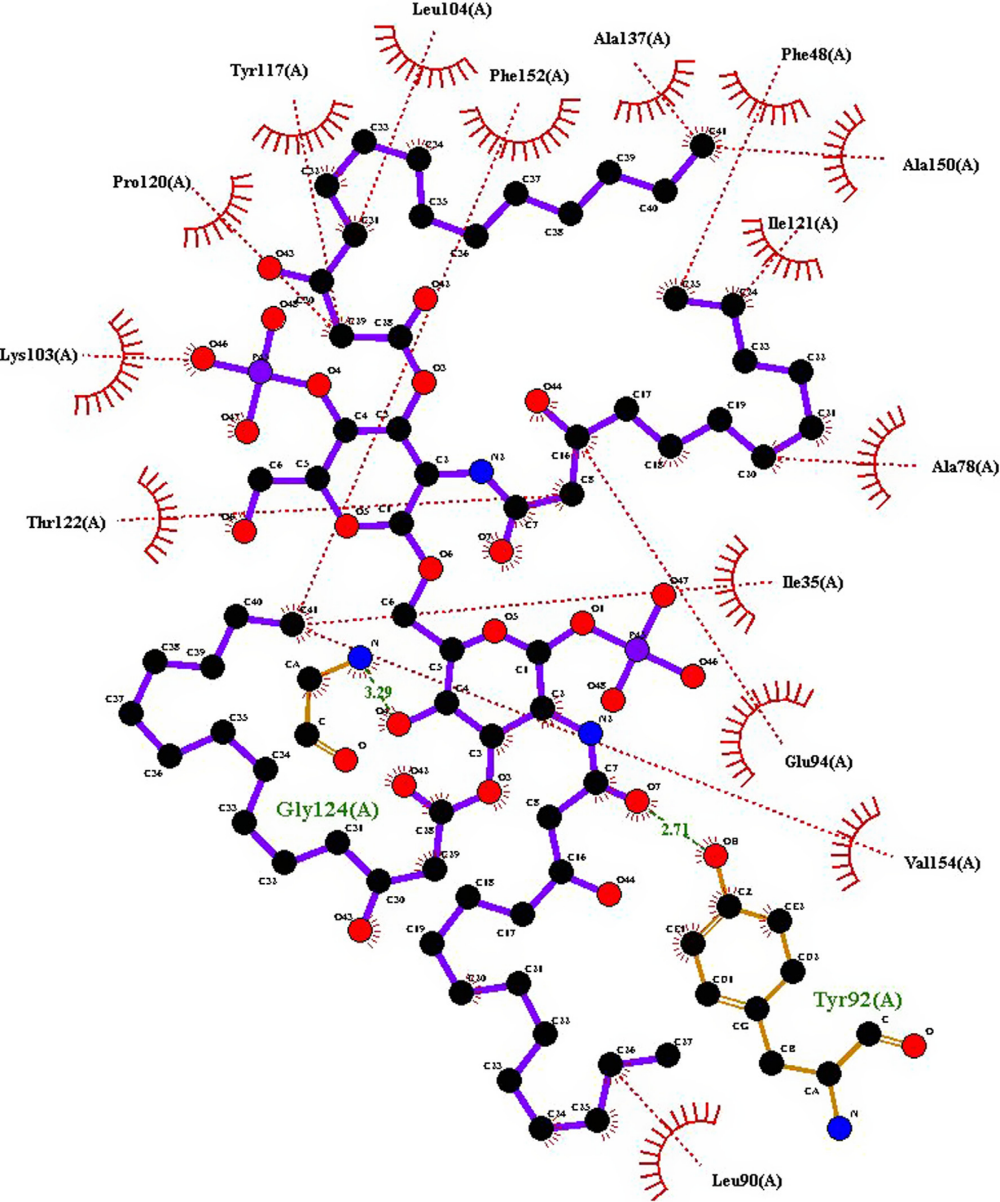
Molecular docking of HN multi-epitope vaccine ligand with TLR4 receptor, view structure on Ligplot. Hydrophobic interactions are depicted as red arcs while, hydrogen bonds are depicted as green dashed lines. Key protein residues are colored black (hydrophobic) or red (hydrogen bonding), while ligand residues are colored blue. Because this is a two-dimensional model, actual distances, bond lengths, and so on were not included.

Top 10 PatchDock results of our receptor TLR4 and ligand vaccine interactions were further refined through FireDock web server. After refinement and scoring FireDock ranked first model which has highest global energy (3.70) was selected to visualize by using Ligplot + as shown in (Fig. 3). Two receptor residues Tyr92 and Gly 124 were interacting with the ligand through the hydrogen bond (green lines) while 15 other receptor residues were interacting through hydrophobic interactions (red dashed lines). So, there are total 17 interactions predicted by the docking of TLR4 and vaccine.

### 3.8 Vaccine construct immune simulation

**Fig. 4:**
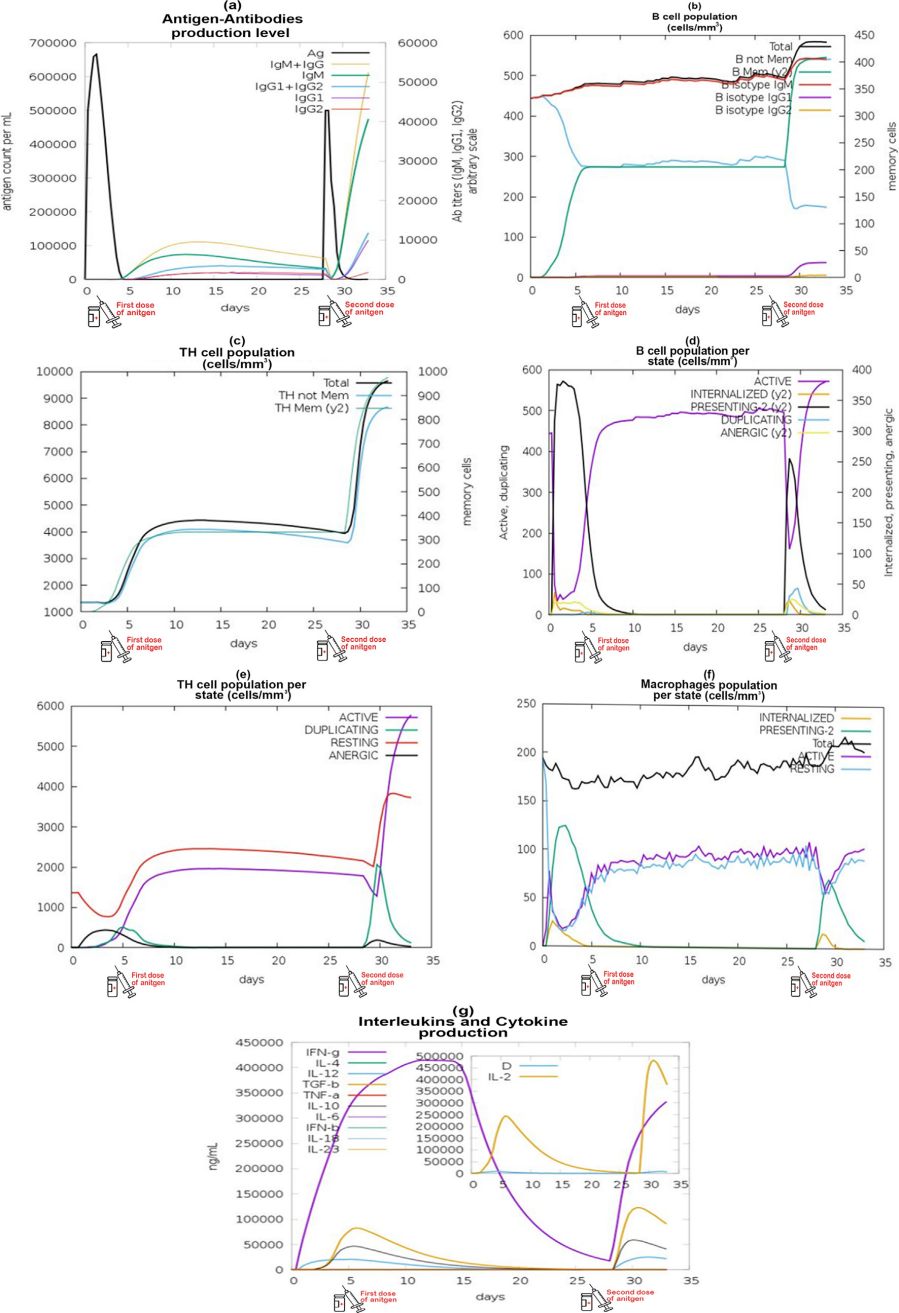
C-ImmSim analysis (a) antibodies generation when exposed to antigen (b) amount of B- cell population; (c) amount of per state plasma B-lymphocytes; (d) during immune response population of helper T- cell; (e) population of antigen exposure of cytotoxic T-cell; (f) macrophage population activity in two subsequent immunological responses; (g) Simpson index (D) was used to compare the production of cytokines and interleukins in different states.

C-IMMSIM immune server generated results in the form of graphs for an *In silico* immunogenic profile analysis of our multi-epitope vaccine. Upon primary immune response antigen (black line) raise to > 600000 counts per ml within the blood. Consequently, antibodies titres (IgM + IgG = yellow, IgM = green, IgG1 + IgG2 = sky blue, IgG1 = purple and IgG2 = red line) were also increased to the scale of 10,000 (Fig. 4**a**). In primary response both B-cells (Fig. 4**b**) and TH cell population (Fig. 4**c**) concentrations were also raised up to the scale of 370 cells/mm^3^ and 400 cells/mm^3^ respectively. In secondary immune response, antigen count per ml again raised which boost the antibodies, B cells and TH cells population to the scale of 50000, 400 cells/mm^3^ and 1000 cells/mm^3^ respectively. The active B-cells mentioned by purple line in graph of Fig. 4**d** and some other types of B-cells like duplicating B-cells (sky blue line) was continuously increased by keeping the memory of vaccine administration during the time interval between two doses. In this graph, the level of inactive B-cells (yellow line) was deepest which was good for our immune simulation analysis. The active TH cells (purple line) increased from day 5 to 10 then their level remained constant till day 27 until the second dose administration (Fig. 4**e**). The number of anergic or inactive TH cells was decreased upon each dose. In Fig. 4**f**, graph increased macrophage activity was also seen with each dose. Fig. 4**g** graph showed that the number of interleukins and cytokines which were also found to be increased within the blood after upon each vaccine dose. The levels of both Interleukin-10 and interleukin-12 increased with first dosage and then upon the second dosage. The level of second dose is higher than the level of first dose. The insert plot showed the development of various epitope-specific dominant clones of IL-2 throughout the time as indicated by an increase in the Simpson index (D). So, we concluded that our designed vaccine may generated a substantial amount of immune response in chicken upon the administration of two booster doses because the secondary immune response is faster and stronger than the primary immune response.

## 4 Discussion

In this study, we constructed and analyzed multi epitope based vaccine against NDV by using several different bioinformatics tools. Additionally, we analyzed immune responses of the vaccine construct through immune simulation. The vaccine construct comprises of T-Cell and B-cell epitopes, as well as appropriate linkers and adjuvant. The adjuvant (gallinacin-1 alpha precursor avian β-defensin) is a 65 amino acid long peptide (Brockus, Jackwood, and Harmon 1998) that functions as an antibacterial agent as well as an immunomodulator (G. Zhang and Sunkara 2014). The avian adjuvant with the first CTL epitope of the HN protein, was linked using a-helix-forming peptide linkers (EAAAK) (Wu, Fan, and Li 2009). Nain et al. (2020) suggested that EAAAK linker increase the bifunctional catalytic activity and decrease the toxicity of protein. Remaining CTL-epitopes were linked with AAY linkers, while HTL epitopes and B-cell epitopes were linked with GPGPG and KK respectively (Nain et al. 2020). Negahdaripour et al. (2018) suggested that linkers bring the construct pH level near to the physiological range.

The designed construct was found to be non-allergenic, antigenic and non-toxic and has 259 amino acids residues (28.18 KDa). Mugunthan et al (2021) have designed vaccine against *Mycoplasma gallisepticum* which was 196 amino acids long. Furthermore, Kar et al. (2020) designed vaccine which even had 422 amino acid residues. As a result, our findings indicate that the size of our vaccine would not be an issue in terms of effectiveness, stability, or expression. The final construct was significantly basic (theoretical PI < 7) (Behmard et al. 2020) indicating that it may offer a sustained contact within the normal pH range. In addition, calculated AI and II indicates its stability (Ikai 1980; W. A. Jensen, Szarka, and White 2019) while negative GRAVY indicated its hydrophilicity, implying strong interactions with water molecules (Chang and Yang 2013).

Molecular docking between immune receptor such as TLR and vaccine was used to investigate in order to ensure the proper transformation of vaccine into the body (Black et al. 2010). The host produces an efficient immune response if a vaccine interacts properly with the target immune cells. Therefore, molecular docking was carried out to examine binding between the MEV and the chicken immune receptor (ChTLR4). ChTLR4 has been extensively studied and researchers found its vital roles in the generation of an innate and adaptive immune response (Nawab et al. 2019). In this work, molecular docking revealed robust interactions between the vaccine and TLR4 for efficient binding. This finding was also supported by the highest ΔG.

The lower the D number, the less diverse the population expression. (Shey et al. 2019). In addition, we discovered an abundance of active immunoglobulins, such as IgG, IgM, and their isotypes, which could be involved in isotype switching. As a result, the reproduced immune response was differentiated by greater rates of helper B-cell and T-cell activity. As a result of these data, we may conclude that our vaccine design can effectively elicit the immune response and provide the foundation for immunization against Newcastle disease virus-related infections.

## 5 Conclusion

This study employs immunoinformatic methods to develop a multi-epitope vaccine against Newcastle disease virus. *In silico* technologies can be used to develop a more effective vaccine in less time and at a cheaper cost. The vaccine was found to be highly immunogenic, non-allergenic, non-toxic, and antigenic with a high affinity for the TLR4 immune receptor. To ensure the stability of the proposed vaccine, molecular dynamics simulation was used, and Molecular Docking investigations confirmed a stable interaction of the vaccines with immune receptors. Furthermore, the simulated immune response displayed a variety of characteristics, including cellular and humoral immune responses, as well as efficient memory cell development. The current study, on the other hand, is the sole result of a computer-based computational technique; to clarify the efficacy and safety of the vaccine, experimental validation is required, which may include the synthesis of vaccine protein with thorough *In vivo* and *In vitro* assessments.

## Funding source

We are thankful to Higher Education Commission (HEC), Pakistan to support this work. The work is done under HEC project Ref. No. 20–11561/NRPU/R&D/HEC/2020.

## Declaration of Competing Interest

The authors declare that they have no known competing financial interests or personal relationships that could have appeared to influence the work reported in this paper.
